# Research priorities in maternal and neonatal health in Africa: results using the Child Health and Nutrition Research Initiative method involving over 900 experts across the continent

**DOI:** 10.12688/aasopenres.13189.1

**Published:** 2021-02-23

**Authors:** Moses Alobo, Charles Mgone, Joy Lawn, Colette Adhiambo, Kerri Wazny, Chinyere Ezeaka, Elizabeth Molyneux, Marleen Temmerman, Pius Okong, Address Malata, Thomas Kariuki, African Academy of Sciences Maternal and Neonatal Health Working Group for Africa

**Affiliations:** 1 African Academy of Sciences, Nairobi, Kenya; 2 Hubert Kairuki University, Dar es Salaam, Tanzania; 3 London School of Hygiene and Tropical Medicine, London, UK; 4 Bloomberg School of Public Health, Johns Hopkins University, Maryland, USA; 5 College of Medicine, University of Lagos/Lagos University Teaching Hospital, Lagos, Nigeria; 6 College of Medicine, University of Malawi, Malawi, Blantyre, Malawi; 7 Aga Khan University, Nairobi, Kenya; 8 Health Service Commission, Kampala, Uganda; 9 Malawi University of Science and Technology,, Thyolo, Malawi

**Keywords:** Maternal health, neonatal health, newborn health, CHNRI

## Abstract

**Background:** Africa will miss the maternal and neonatal health (MNH) Sustainable Development Goals (SDGs) targets if the current trajectory is followed. The African Academy of Sciences has formed an expert maternal and newborn health group to discuss actions to improve MNH SDG targets. The team, among other recommendations, chose to implement an MNH research prioritization exercise for Africa covering four grand challenge areas.

**Methods: **The team used the Child Health and Nutrition Research Initiative (CHNRI) research prioritization method to identify research priorities in maternal and newborn health in Africa. From 609 research options, a ranking of the top 46 research questions was achieved. Research priority scores and agreement statistics were calculated, with sub-analysis possible for the regions of East Africa, West Africa and those living out of the continent.

**Results:** The top research priorities generally fell into (i) improving identification of high-risk mothers and newborns, or diagnosis of high-risk conditions in mothers and newborns to improve health outcomes; (ii) improving access to treatment through improving incentives to attract and retain skilled health workers in remote, rural areas, improving emergency transport, and assessing health systems' readiness; and (iii) improving uptake of proven existing interventions such as Kangaroo Mother Care.

**Conclusions:** The research priorities emphasized building interventions that improved access to quality healthcare in the lowest possible units of the provision of MNH interventions. The lists prioritized participation of communities in delivering MNH interventions. The current burden of disease from MNCH in Africa aligns well with the list of priorities listed from this exercise but provides extra insights into current needs by African practitioners.

The MNCH Africa expert group believes that the recommendations from this work should be implemented by multisectoral teams as soon as possible to provide adequate lead time for results of the succeeding programmes to be seen before 2030.

## Introduction

The deceleration in average annual rates of reduction of maternal mortality ^1^ and neonatal mortality indicators ^2^ signal a need to do more to achieve the Sustainable Development Goals (SDG) targets. Maternal and neonatal health (MNH) global burden of disease statistics remains alarmingly high, with a global burden of 5.4 million deaths, including 2.5 million newborn deaths ^3^, 2.6 million stillbirths ^4^, and 0.3 million maternal deaths ^1^. With just over a decade to meet the SDGs, Africa, with only 13% of the world's population, carries more than half of this burden with 2.3 million deaths per year. Based on current trends, most sub-Saharan African countries ^5^ will not meet the SDG target of 12 or fewer newborn deaths per 1,000 births and are also at risk of missing targets for maternal mortality reduction ^5^. The current COVID-19 outbreak could indirectly result in an additional 1,157,000 child deaths and 56,700 maternal deaths according to modelling data under a most severe scenario of 39.3–51.9% reduction in coverage rates ^6^. There are calls to national programmes to provide core maternal and child interventions, even with the risk of COVID-19 transmission ^7^ and also build catch-up phases for the indicators.

This scenario calls for urgent actions to address MNH challenges, especially in low- and middle-income countries (LMICs) ^8^, which face challenges due to low investments in health interventions. There is a need for Africa to prioritize areas for research and development in MNH in order to appropriately utilize the limited resources ^9^ available for healthcare.

In a 2019 report ^5^, MNH experts on the African continent convened by the African Academy of Sciences (AAS) and the U.K. Academy of Medical Sciences (UK AMS) listed four grand challenges for MNH in Africa with details on specific areas in implementation and discovery science with additional cross-cutting areas of importance. The four grand challenges included: (i) better care during pregnancy; (ii) better care at birth; (iii) better postnatal care for women and newborns; and (iv) better hospital care for sick newborns. The report recognizes the critical role a research and development priorities list for MNH in Africa could play in realizing the SDG goals.

Considering this, a MNH research priority setting exercise was developed, including more than 900 experts, between March and October 2019, as described below. The work aimed to deliver an MNH research priority list for Africa, covering the four grand challenge areas in which science and research in this field are crucial in accelerating implementation and developing innovations. The expert group believes the recommendations from this work should be implemented as soon as possible to provide an adequate lead time for results of the succeeding programmes to be seen before 2030.

## Methods

The group used the Child Health and Nutrition Initiative (CHNRI) method ^10^ (first used in 2008) as the scheme for the research prioritization exercise. It is currently the dominant method in health research prioritization. It has been used over 100 times with constant updates ^11^ to set global ^10^, national ^12^, and regional ^13^ research priorities in areas ranging from maternal, newborn ^12^, child health and nutrition ^14^, in humanitarian settings ^15^, sexual health, disability, and dementia ^16^. CHNRI uses the principles of the *wisdom of the crowds* to collect systematically and transparently score research options against pre-set criteria in a particular field ^17, 18^.

We followed the following steps to undertake this initiative:

i. A Steering Committee defined the context of the exercise
ii. MNH experts were invited to participate and submit research options within the context of interest
iii. Similar research options received were consolidated into singular priorities
iv. External stakeholders were invited to set different weights on the criteria for scoring
v. Research options were scored against pre-set criteria by a larger group of experts
vi. Research questions were ranked, and the Research Priority Scores and the agreement statistics for each priority were calculated

The Steering Committee constituted by the Grand Challenges Africa programme at the African Academy of Sciences oversaw the CHNRI exercise in 2019 for MNH in Africa. An independent CHNRI expert (KW) provided input on the methodology. 

### *i. The context of the exercise is defined* 

The exercise aimed to identify research priorities in maternal and newborn morbidity, mortality, and disability in Africa using the CHNRI exercise. The grand challenge areas for MNH identified from a previous workshop ^5^ conducted in September 2018 identified four critical 'grand challenges' that need addressing, which formed the basis of the research prioritization exercise. These grand challenges were: (i) better care during pregnancy; (ii) better care at birth; (iii) better postnatal care for women and their infants; and, (iv) better hospital care of sick newborns. Each grand challenge was also described under the continuum of research development, namely (i) description, (ii) discovery, (iii) development and (iv) delivery. This proposed framework for systematic listing of research ideas in health research takes into account the listing of health research areas and the depth of the recommended research ideas ^17^.

### *ii. Experts are invited to participate and submit research options within the context of interest* 

Over 700 ^1^ experts, identified through a database held by the AAS, and through a literature review and snowballing, were invited to submit research ideas. Research ideas were submitted via an online survey from 251 experts using Survey Monkey (see *Extended data* ^19^). Experts were asked to submit up to four ideas each within any of the four grand challenge domains. We received a total of 609 research ideas. 

### *iii. Research options are consolidated* 

The long list of 609 research questions was consolidated into 403 research options by removing duplicates and combining similar ideas. The research team then classified the research options into the 4D categories, 'discovery, development, delivery, or description' (defined in Box 1), intended to cover all possible types of research questions ^17^.

**Box 1. Definition of the 'Four Ds'**

**Description** – research to assess the burden or risk factors for the problem (e.g. disease).

**Discovery** – research to develop (or discover) new interventions or innovations.

**Development** – research to improve upon existing interventions.

**Delivery** – research to optimize the health status of the population using existing interventions (e.g. operational research, cost effectiveness, policy).

Members of the Steering Committee evaluated the consolidated list of research options, refined the wording further, and merged the research options into a list of 281. This list was then scored independently from 1 to 5 (with 1 representing a less important research option, and 5 representing an extremely important research option). An average was calculated, and a cut-off score of > 4.25 was used that selected 46 top research options to be presented for scoring by the larger group of experts (see *Underlying data* ^19^). As scoring for the CHNRI exercise can be onerous, it was decided to limit the number of research options scored by the larger group of experts in order to maximize response rate and reduce scorer fatigue. 

### *iv. External stakeholders weigh the criteria* 

A workshop was held in June 2019, which presented the criteria ( Table 1) to stakeholders, including representatives from the public and private sector, donors, civil society organizations, clinicians, and academics. A total of 42 stakeholders determined by availability but balanced by region, discipline, expertise, and gender participated in the weighting exercise. Stakeholders were asked to rank the criteria from 1 to 4 (with 1 being the most important, and 4 being the least important).

**Table 1.** Description of CHNRI criteria and weights.

Criterion	Sub-Questions	Weight
Disease burden reduction	i. Are the results of the research likely to reduce the burden of maternal or neonatal **mortality**? ii. Are the results of the research likely to reduce the burden of maternal or neonatal **morbidity**? iii. Are the results of the research likely to reduce the burden of maternal or neonatal **disability**?	0.66
Answerability	i. Is the research question well-framed, with well-defined endpoints? ii. Is it likely that, in the context of interest, there will be sufficient capacity to carry out this research? iii. Do you think the research could obtain ethical approval without major concerns?	1.10
Potential Impact and Equity	i. Will the results of this research fill an important knowledge gap and result in a genuine, innovative improvement over existing business-as-usual? ii. Are the results from this research likely to shape future planning and implementation? iii. Does this research impact the lives of those most vulnerable (e.g. those in lower wealth quintiles, those who are most marginalized, or those in hard-to-reach areas)	0.94
Deliverability	i. Taking into account the level of difficulty with delivery of the potential intervention or delivery strategy (for example, need for change of attitudes and beliefs, supervision, transport infrastructure), would you say that this intervention or delivery strategy will be ***deliverable*** and scalable within the context of interest? ii. Taking into account the resources available to implement the intervention, would you say that the intervention or delivery strategy would be ***affordable and cost effective*** within the context of interest? iii. Would government capacity and partnership be essential to ensure the intervention or delivery strategy would be ***sustainable***?	1.30

An average across each criterion was calculated, and converted to a weight using the following formula (demonstrated for criterion 1):

weight(criterion 1)= [(∑scores criterion 1ncriterion 1)10/]*4

Weights for each criterion can be found in Table 1.

### *v. Research options are scored against pre-set criteria by the large group of experts* 

Survey Monkey was used to circulate the list of 46 research questions to the wider group of experts. The experts scored the questions against pre-set criteria, developed by the independent consultant in consultation with the Steering Committee ( Table 1). The criteria were phrased as yes or no questions; experts were given the choice of answering yes, no, or I don't know, or uninformed. 

A total of 319 experts from across all regions (North, South, East, and West) in Africa scored the research questions in the 2-hour survey where questions were randomized and presented to scorers in a different order. Invited experts were top publishing authors from each country in MNCH from 2015 to 2019. Table 2 contains individual and demographic characteristics for each scorer, including country of residence, gender, age, and area of specialization. Duplicate responses were removed and scorers included in the analysis if they scored at least one full research question. A total of 195 scorers filled the survey completely (e.g. entered a score for every criterion for each research question).

**Table 2.** Individual and demographic characteristics of experts who participated in scoring.

Description	Category	No. of Participants
Gender	Male	144
	Female	115
	Not specified	60
Age	Under 30	8
	30–39	84
	40 – 49	88
	50 – 59	52
	60 – 69	23
	70 +	4
	Not specified	60
Sector	Academia & Teaching	137
	Research	55
	Healthcare Provision	22
	N.G.O.	18
	Government	9
	Other or unspecified	78
Region in of residence in Africa	North Africa	2
	West Africa	56
	East Africa	82
	Central Africa	6
	Southern Africa	34
	Do not reside in Africa	79
	Not specified	60

### *vi. Research questions are ranked using Research Priority Score and agreement calculated* 

The data team calculated the Research Priority Scores (RPS) and the Average Expert Agreement (AEA) for each research question using Excel (Version 16.45) (Excel for Mac). The RPS, which is a mean score across the number of scorers for each research question, was calculated both weighted and unweighted. In the weighted scores, the RPS was first calculated within each criterion, then the stakeholder weights were applied to the within-criteria RPS, and a final RPS (across criteria) was calculated as an average score across criteria. Scores above 1.0 are therefore possible under the weighted calculations.

The AEA is the mode (i.e. the proportion of experts scoring the most common answer) and is calculated on the unweighted scores. 

We conducted several sub-analyses including research priorities in MNH for East Africa; research priorities for West Africa; research priorities among international experts (e.g., those who do not reside in Africa); research priorities within each 'grand challenge' category; and, research priorities within each 4D category.

## Results

The full list of research priorities overall and within each subgroup can be found in the Supplementary File.

### Continental-level results

The top ten research priorities overall are presented in Table 3, alongside their weighted scores within each criterion, RPS and AEA. The RPS ranges from 0.89 to 0.76 and AEA ranged from 0.85 to 0.66. Higher AEA correlated with higher RPS, which indicates that experts agreed on the research priorities that scored highest, and those that had lower scores may have had a mix of high and lower scores.

**Table 3.** Top 10 research questions (across Africa), displayed with RPS, AEA, grand challenges category, and 4D category.

Rank	Research Question	4D Category	Grand Challenges Category	Disease Burden	Answerability	Impact/ Equity	Deliverability	RPS	AEA
**1**	Evaluate the impact of empowering mothers to recognise danger signs on newborn health outcomes	Delivery	Better care during pregnancy	0.60	0.98	0.83	1.15	0.89	0.85
**2**	Develop strategies to improve detection of pregnancy-induced hypertension at the primary care¬†level	Development	Better care during pregnancy	0.61	0.97	0.82	1.13	0.88	0.84
**3**	What are the optimal methods to attract and retain skilled birth attendants in remote, rural areas?	Delivery	Better Care at Birth	0.60	0.97	0.85	1.10	0.88	0.84
**4**	Assess uptake of best practices to reduce neonatal sepsis (including rational use of prophylactic antibiotics, clean birth environment) in prevention of neonatal sepsis¬†	Delivery	Better hospital care of sick newborns	0.60	0.99	0.81	1.11	0.88	0.83
**5**	Develop methods to enhance quality of care for sick newborns through early identification and appropriate therapeutic measures for the management of neonatal sepsis	Delivery	Better hospital care of sick newborns	0.62	0.96	0.83	1.08	0.87	0.82
**6**	Develop and evaluate strategies for improved utilization¬†of Kangaroo Mother Care (KMC)¬†at the community level	Delivery	Better postnatal care for women and their newborns	0.58	0.98	0.81	1.10	0.87	0.82
**7**	Assess uptake of best practices in hospital care of the preterm infants, evaluate coverage of Kangaroo Mother Care (KMC) and determine the barriers and facilitators for its uptake	Delivery	Better hospital care of sick newborns	0.59	0.97	0.80	1.11	0.87	0.81
**8**	Design and test new algorithms and point of care diagnostic tests for sepsis (including postnatal sepsis) for mothers and babies, care of sick newborns and children in the face of emergencies and epidemics	Discovery	Better Care at Birth	0.61	0.92	0.86	1.07	0.86	0.82
**9**	Design and test innovative solutions to improve access to emergency transport in the community (e.g. ride share, local transport)	Delivery	Better Care at Birth	0.60	0.95	0.83	1.04	0.86	0.82
**10**	Identify the most effective and affordable tools to be deployed in the lowest level of health facilities to provide lifesaving care to sick neonates in African countries (e.g. availability of oxygen, functional newborn units, etc.)	Development	Better hospital care of sick newborns	0.61	0.93	0.85	1.05	0.86	0.82

The top priority overall was 'to empower mothers to recognize danger signs and evaluate the impact on newborn health outcomes'. The top ten research priorities overall ( Table 3), focused on detection or diagnosis to improve treatment outcomes (#s 1, 2, 5, and 8), improvements in access to care, through improved staffing (#3), access to emergency transport (#4), or access to affordable tools at the lowest level of health care (#10). Improving the quality of care was also a focus (#3, 4, and 5), as was kangaroo mother care at both the community- (#6) and facility-level (#7).

Seven of the top ten research priorities were categorized as 'delivery' research, two were classified as development, and one as discovery. This is in line with the distribution of other CHNRI exercises ^11^. Three of the top ten research priorities were within the grand challenge 'Better care at birth,' two were in 'Better care during pregnancy,' four were in 'Better hospital care of sick newborns,' and one was in 'better postnatal care for women and their newborns.'

### Subgroup analyses

Subgroup analyses were conducted for West Africa, East Africa, and International scorers. A comparison of ranks for each research priority between these groups and the overall (continental) scores can be found in Table 4. Ranks within the subgroups that are over a ten-point deviation from the continental ranks are highlighted in orange. Ranks that are over a five-point deviation from the continental ranks are highlighted in yellow.

**Table 4.** Comparison of ranks within subgroup analyses.

Continental Rank	Research Question	4D Category	Grand Challenges Category	East Africa Rank	West Africa Rank	International Rank
**1**	Evaluate the impact of empowering mothers to recognise danger signs on newborn health outcomes	Delivery	Better care during pregnancy	**1**	**1**	**19**
**2**	Develop strategies to improve detection of pregnancy-induced hypertension at the primary care¬†level	Development	Better care during pregnancy	**3**	**2**	**3**
**3**	What are the optimal methods to attract and retain skilled birth attendants in remote, rural areas?	Delivery	Better Care at Birth	**6**	**7**	**1**
**4**	Assess uptake of best practices to reduce neonatal sepsis (including rational use of prophylactic antibiotics, clean birth environment) in prevention of neonatal sepsis¬†	Delivery	Better hospital care of sick newborns	**2**	**23**	**6**
**5**	Develop methods to enhance quality of care for sick newborns through early identification and appropriate therapeutic measures for the management of neonatal sepsis	Delivery	Better hospital care of sick newborns	**4**	**3**	**9**
**6**	Develop and evaluate strategies for improved utilization¬†of Kangaroo Mother Care (KMC)¬†at the community level	Delivery	Better postnatal care for women and their newborns	**10**	**22**	**2**
**7**	Assess uptake of best practices in hospital care of the preterm infants, evaluate coverage of Kangaroo Mother Care (KMC) and determine the barriers and facilitators for its uptake	Delivery	Better hospital care of sick newborns	**5**	**6**	**13**
**8**	Design and test new algorithms and point of care diagnostic tests for sepsis (including postnatal sepsis) for mothers and babies, care of sick newborns and children in the face of emergencies and epidemics	Discovery	Better Care at Birth	**8**	**18**	**5**
**9**	Design and test innovative solutions to improve access to emergency transport in the community (e.g. ride share, local transport)	Delivery	Better Care at Birth	**17**	**9**	**4**
**10**	Identify the most effective and affordable tools to be deployed in the lowest level of health facilities to provide lifesaving care to sick neonates in African countries (e.g. availability of oxygen, functional newborn units, etc.)	Development	Better hospital care of sick newborns	**15**	**15**	**7**

***West Africa***. When limited to West Africa, the RPS ranged from 0.98 to 0.85 and the AEA ranged from 0.99 to 0.77. The top ten research priorities are presented in Table 5. The top research priority was 'to evaluate the impact of mothers recognizing danger signs on newborn health outcomes'. There was a pronounced focus on developing improved diagnostics, algorithms, or tools to identify at-risk mothers or infants more effectively (#s 1, 2, 3, 4, and 8), identifying ways to improve access to treatment through availability of skilled health workers in remote and rural areas (by identifying incentives for them) (#7), identifying innovative ways to improve access to emergency transport (#9), and assessing the health systems' readiness to handle emergency obstetric complications (#10). Research to improve access to antenatal nutrition and to demonstrate its impact on intrauterine growth was also prioritized (#5). West Africa prioritized antenatal nutrition (#5) which was otherwise considered as #26 ^th^ overall on the continental priorities.

**Table 5.** Top 10 research priorities maternal and newborn health in West Africa.

Rank	Research Question	4D Category	Grand Challenges Category	Disease Burden	Answerability	Impact/ Equity	Deliverability	RPS	AEA
**1**	Evaluate the impact of empowering mothers to recognise danger signs on newborn health outcomes	Delivery	Better care during pregnancy	0.66	1.10	0.94	1.24	0.98	0.99
**2**	Develop strategies to improve detection of pregnancy-induced hypertension at the primary care¬†level	Development	Better care during pregnancy	0.65	1.08	0.87	1.23	0.96	0.96
**3**	Develop methods to enhance quality of care for sick newborns through early identification and appropriate therapeutic measures for the management of neonatal sepsis	Delivery	Better hospital care of sick newborns	0.66	1.04	0.94	1.18	0.96	0.95
**4**	Develop improved algorithms to risk stratify women in antenatal care to facilitate earlier referral to higher levels of care	Discovery	Better care during pregnancy	0.65	1.04	0.92	1.20	0.95	0.95
**5**	Evaluate the impact of improving maternal nutrition including micronutrients during pregnancy and its impact on intra-uterine¬†growth development and other neonatal outcomes using large-scale implementation studies	Development	Better care during pregnancy	0.65	1.02	0.93	1.18	0.94	0.93
**6**	Assess uptake of best practices in hospital care of the preterm infants, evaluate coverage of Kangaroo Mother Care (KMC) and determine the barriers and facilitators for its uptake	Delivery	Better hospital care of sick newborns	0.64	1.04	0.87	1.21	0.94	0.90
**7**	What are the optimal methods to attract and retain skilled birth attendants in remote, rural areas?	Delivery	Better Care at Birth	0.63	1.04	0.92	1.18	0.94	0.93
**8**	Determine the most cost effective way to identify pregnant mothers at risk of adverse pregnancy outcomes during their antenatal care for better care during pregnancy (prevention) and during the intrapartum period (labour management)	Delivery	Better care during pregnancy	0.64	1.02	0.90	1.19	0.94	0.91
**9**	Design and test innovative solutions to improve access to emergency transport in the community (e.g. ride share, local transport)	Delivery	Better Care at Birth	0.66	1.03	0.91	1.15	0.94	0.93
**10**	Assess health systems' readiness in handling emergency obstetric complications	Delivery	Better Care at Birth	0.64	1.00	0.92	1.18	0.94	0.92

***East Africa***. The top ten research questions for scorers from East Africa are presented in Table 6. RPS for all research questions were scored highly and ranged from 1.19 to 1.03 and AEA ranged from 0.93 to 0.68. The top research priority is shared with that of the continent and West Africa (evaluating the impact of mothers recognizing danger signs on newborn health outcomes). Use of risk stratification, improvement of algorithms, and improving methods for detection and diagnoses of high-risk maternal and newborn conditions are also a priority in East Africa (#s 1, 3, 4, 7, 8). Research priorities involving improving management of newborns, through improving uptake of essential newborn care (#9) and Kangaroo Mother Care in facilities (#5) and communities (# 10) were prioritized.

**Table 6.** Top 10 research priorities in maternal and newborn health in East Africa.

Rank	Research Question	4D Category	Grand Challenges Category	Disease Burden	Answerability	Impact/ Equity	Deliverability	RPS	AEA
**1**	Evaluate the impact of empowering mothers to recognise danger signs on newborn health outcomes	Delivery	Better care during pregnancy	0.64	1.03	0.85	1.24	1.19	0.93
**2**	Assess uptake of best practices to reduce neonatal sepsis (including rational use of prophylactic antibiotics, clean birth environment) in prevention of neonatal sepsis¬†	Delivery	Better hospital care of sick newborns	0.63	1.01	0.84	1.20	1.18	0.90
**3**	Develop strategies to improve detection of pregnancy-induced hypertension at the primary care¬†level	Development	Better care during pregnancy	0.64	0.96	0.89	1.17	1.17	0.89
**4**	Develop methods to enhance quality of care for sick newborns through early identification and appropriate therapeutic measures for the management of neonatal sepsis	Delivery	Better hospital care of sick newborns	0.66	0.97	0.84	1.16	1.17	0.89
**5**	Assess uptake of best practices in hospital care of the preterm infants, evaluate coverage of Kangaroo Mother Care (KMC) and determine the barriers and facilitators for its uptake	Delivery	Better hospital care of sick newborns	0.62	1.02	0.84	1.14	1.16	0.89
**6**	What are the optimal methods to attract and retain skilled birth attendants in remote, rural areas?	Delivery	Better Care at Birth	0.61	0.99	0.88	1.11	1.16	0.87
**7**	Develop improved algorithms to risk stratify women in antenatal care to facilitate earlier referral to higher levels of care	Discovery	Better care during pregnancy	0.60	1.00	0.84	1.14	1.16	0.87
**8**	Design and test new algorithms and point of care diagnostic tests for sepsis (including postnatal sepsis) for mothers and babies, care of sick newborns and children in the face of emergencies and epidemics	Discovery	Better Care at Birth	0.64	0.95	0.89	1.08	1.15	0.86
**9**	Develop and evaluate impact of strategies to improve uptake of essential newborn care on neonatal outcomes	Delivery	Better hospital care of sick newborns	0.65	0.94	0.84	1.14	1.15	0.86
**10**	Develop and evaluate strategies for improved utilization¬†of Kangaroo Mother Care (KMC)¬†at the community level	Delivery	Better postnatal care for women and their newborns	0.61	0.99	0.83	1.11	1.15	0.86

***International scorers***. An additional subgroup analysis was conducted for those residing outside Africa, as a previous CHNRI exercise ^20^ has suggested there may be different research priorities for this group. The top 10 research priorities for this group are displayed in Table 7. RPS ranged from 0.84 to 0.63 and AEA ranged from 0.78 to 0.52, indicating substantially less agreement among this group than within the other subgroup analyses.

**Table 7.** Top 10 research priorities in maternal and newborn health from International Scorers.

Rank	Research Question	4D Category	Grand Challenges Category	Disease Burden	Answerability	Impact/ Equity	Deliverability	RPS	AEA
**1**	What are the optimal methods to attract and retain skilled birth attendants in remote, rural areas?	Delivery	Better Care at Birth	0.57	0.92	0.84	1.03	0.84	0.78
**2**	Develop and evaluate strategies for improved utilization¬†of Kangaroo Mother Care (KMC)¬†at the community level	Delivery	Better postnatal care for women and their newborns	0.54	0.94	0.74	1.04	0.82	0.71
**3**	Develop strategies to improve detection of pregnancy-induced hypertension at the primary care¬†level	Development	Better care during pregnancy	0.55	0.90	0.74	1.05	0.81	0.71
**4**	Design and test innovative solutions to improve access to emergency transport in the community (e.g. ride share, local transport)	Delivery	Better Care at Birth	0.59	0.91	0.78	0.95	0.81	0.73
**5**	Design and test new algorithms and point of care diagnostic tests for sepsis (including postnatal sepsis) for mothers and babies, care of sick newborns and children in the face of emergencies and epidemics	Discovery	Better Care at Birth	0.57	0.86	0.76	1.02	0.80	0.70
**6**	Assess uptake of best practices to reduce neonatal sepsis (including rational use of prophylactic antibiotics, clean birth environment) in prevention of neonatal sepsis¬†	Delivery	Better hospital care of sick newborns	0.55	0.91	0.72	0.99	0.79	0.70
**7**	Identify the most effective and affordable tools to be deployed in the lowest level of health facilities to provide lifesaving care to sick neonates in African countries (e.g. availability of oxygen, functional newborn units, etc.)	Development	Better hospital care of sick newborns	0.54	0.82	0.79	1.01	0.79	0.72
**8**	Conduct large-scale implementation research of interventions that have been demonstrated to improve quality of care during labour	Delivery	Better Care at Birth	0.57	0.81	0.76	1.00	0.79	0.72
**9**	Develop methods to enhance quality of care for sick newborns through early identification and appropriate therapeutic measures for the management of neonatal sepsis	Delivery	Better hospital care of sick newborns	0.58	0.87	0.75	0.94	0.78	0.68
**10**	Determine the impact of 'skilling drills' on obstetricians, general practitioners, and midwives in handling obstetric emergencies in a timely manner through simulations and drills	Development	Better Care at Birth	0.53	0.88	0.72	0.99	0.78	0.64

The top research question in this subgroup was 'to identify optimal methods to attract and retain skilled birth attendants in remote, rural areas'. Research priorities focused on improving access and quality of care featured in half of the top ten questions (#s 1, 4, 8, 9, 10). This group also prioritized detection of high risk maternal or newborn health conditions (#s 3, 5, 6, 9) and improving management of newborns (#2, 7). 

## Discussion

Africa is a diverse continent geographically and politically, the levels of health financing, socio-economic categories, cultures and practices, along with the strategies that shape each country's health delivery systems also differ and in turn, affect the MNH SDG targets. Health systems and contextual factors alongside MNH service delivery interventions, therefore, need to be addressed ^21^ if we are to achieve the MNH SDG's. African MNH experts and practitioners work in this environment, and therefore, it is important to recognize their collective voice when putting together research priorities to support the drive to achieve the SDG's. The MNH group at the African Academy of Sciences believe that this is the most rigorous process yet to highlight the MNH research and development priorities for Africa with the participation of over 900 MNH experts and practitioners.

Of importance, the June 2019 MNH expert convening recommended; deliverability, answerability, potential impact and equity, and disease burden reduction as primary criteria for scoring and weighting the collected priorities. We should, therefore read the results of this MNH prioritization exercise through this lens. 

The results of the survey strongly prioritize 'delivery' options suggesting a need for innovations that can integrate current interventions into existing MNCH primary care and community level systems. For example, the top research question, 'What are the optimal methods to attract and retain skilled birth attendants in remote, rural areas?' requires us to investigate solutions that can constantly provide quality health care workers to the lowest units of MNCH service provision. This need is again expressed in priority number 3 and 9 – 'Develop strategies to improve detection of pregnancy-induced hypertension at the primary care level', and 'Identify the most effective and affordable tools to be deployed in the lowest level of health facilities to provide lifesaving care to sick neonates in African countries (e.g. availability of oxygen, functional newborn units, etc.)'. Priority number 14 and 15 related to primary health care settings too. Strengthening MNCH at the primary healthcare level ^22^ remains a priority in the SDG though some actors propose a shift in focus from strengthening the front-line of service delivery, to changes at the meso-level of sub-district and district decision-making ^23, 24^ for sustainable change at the primary healthcare level. rom 

The survey results emphasize research and development (R&D) for community-based interventions through the selection of three priorities focussing on improving knowledge on newborn danger signs by mothers, improving utilization of Kangaroo Mother Care and innovations that access emergency transport in the community. Implementation of effective community-based primary health care innovations are needed at scale in LMICs ^25^ but most importantly R&D should support these efforts. The selected priorities suggest that mothers and their support community, in the context of cultural and social factors, should be adequately informed of key critical information as they nurse their babies. On the other hand, known interventions with certified benefit need to be rolled out and embraced within communities. R&D is needed to provide sustainable delivery of programmes that surmount infrastructure and access to health provider challenges.

MNH subject areas in the top 15 priorities from this work lists pregnancy-induced hypertension, high-risk pregnant mothers, maternal bacterial infections, neonatal sepsis, low birth weight babies, quality care during labour, perinatal care of the newborn, and referral systems for mothers and babies during emergencies. Health factsheets state that nearly 75% of all maternal deaths in Africa are from severe bleeding (mostly bleeding after childbirth), infections (usually after childbirth), high blood pressure during pregnancy (pre-eclampsia and eclampsia), complications from delivery and unsafe abortion ^26^. Leading causes of death among children under five are preterm birth complications, acute respiratory infections, intrapartum-related complications, congenital anomalies and diarrhoea ^3^. R&D efforts should align with these needs to assist practitioners in achieving better maternal and neonatal outcomes. There is a need for better diagnostics and detection of high-risk newborn and maternal conditions to allow for these conditions to be managed within a slowly reacting healthcare system ^27– 29^. It is important to emphasize that maternal mortality should be considered as a human rights issue, to strengthen the African system's jurisprudence and legal frameworks ^30, 31^ including multisectoral participation of interest groups like human rights non-governmental organizations ^31^ for us to achieve the desired outputs.

Analyzing health indicators in small geographic units aids the identification of hotspots where coverage lags behind neighboring areas ^32^. Likewise, it is expected that researchers' priorities will be influenced by their geographical experiences. There is suspicion that the lower exclusive breastfeeding rates and the higher stunting prevalence rates in West Africa ^32^ could be the drivers of the priority for maternal nutrition in this survey. Multiple micronutrient supplementation during pregnancy and iron folate programmes are therefore a priority for West Africa. Supportive research measures on any pending issues are required for the WHO recommendations put out in 2020 ^33^ on multiple micronutrient supplements during pregnancy.

It is instructive to note that the top continental priority in MNH from this exercise was similar in East Africa and West but ranked 19 ^th^ among international participants. There is evidence from other CHNRI prioritization exercises that international participants sometimes have discordance ^20^ with local scientists' priorities. The international participants' priorities had more alignment with the East African participants than the West African participants. More research would need to be done to establish why this is so. There is a need to align with groups within Africa when designing MNCH programmes on the continent.

To the best of our knowledge, this work is the most rigorous work on MNH research priorities for Africa yet. Despite our best efforts, we cannot rule out the possibilities of bias from our results. The AAS/AESA built a database of about 8,000 MNH commentators from peer-reviewed journals who were invited by email to participate in this survey. Only 10% of this number responded and interacted with the MNH working group for Africa. This provides for a selection bias with only those driven to respond able to complete the 2-hour survey.

However, we aimed to reduce bias by randomizing the order of questions presented to each participant, and by evenly distributing the potential impact of scorer fatigue amongst the research questions. Higher agreement rates between participants are seen for high ranking priorities compared to low ranking priorities. This indicates that there was agreement that the highly ranked priorities are important, but disagreement on the importance of those that ranked poorly. We had hoped to reach sample size for all regions of Africa, but only managed to do a sub-analysis for East Africa, West Africa and the diaspora commentators. We would also consider this as a 'point in time exercise' meaning that the results are only translatable to the time when the priorities were collected; this is especially pertinent due to potential impacts of COVID-19 and potential health and economic disruptions that may occur as a result. A repeat exercise would be required if implementation is delayed.

## Conclusion

This work establishes a maternal and neonatal health research priority list for Africa, covering the four grand challenge areas, in which science and research are crucial in accelerating implementation and developing innovations. The expert group believes that the recommendations from this work are a representation of real-world issues as expressed by researchers and practitioners on the African continent. These priority research questions need to be answered if we are to have a chance to achieve the SDG targets. They also need to be implemented soon to provide an adequate lead time for these proposals to be incorporated into action and results to be seen before 2030.

The African Academy of Sciences through the Grand Challenges Africa and working with partners has implemented a maternal and neonatal healthcare innovations support programme based on these priorities. In addition to this, there is ongoing work on MNH research mapping exercise for Africa which should form a suitable base for the development of an MNH research network. Such a network should be able to foster mentorship programmes in this area, greater coordination and delivery of priorities. There will also be an opportunity for MNH Africa leadership to develop, greater gender representation in MNH science and a consolidated platform for bridging the science policy gap in MNH.

The advent of the pandemic in 2020 on the African continent brought an unprecedented challenge to MNH services and delivery. It is still early days, but anecdotal information points to reductions in MNH indicators due to changes to health seeking behaviour coupled by increased inefficiencies into an already strained health system as COVID-19 status becomes a requirement to access certain services. This is, therefore, a call to action for capable multisectoral organizations to focus on these priority areas as well as institute a catch-up phase for MNH indicators especially if we are to achieve the SDG targets in 2030.

## Data availability

### Underlying data

Open Science Framework: Research priorities in maternal and neonatal health in Africa: results using the Child Health and Nutrition Initiative method involving over 900 experts across the continent, https://doi.org/10.17605/OSF.IO/38GTU ^19^.

This project contains the following underlying data files:

- AAS - CHNRI - Results .xlsx (table lists the ranked final 46 priorities and describes their category according to whether they are description, discovery, development or delivery research focussed options. The table also categorises the questions according to their grand challenge focus area - i.e. better care during pregnancy, better care at birth, better hospital care for sick newborns and better postnatal care for women and their newborns)
- CHNRI Criteria FINAL 2019 June.docx
- Cleaning and evolution of questions CHNRI exercise MNH research options for Africa 2019.xlsx (table listing, in an unranked manner, all original 608 options, the cleaned and condensed 281 options and the final unranked 46 research options)
- Ranked long list of all the priorities.docx

### Extended data

Open Science Framework: Research priorities in maternal and neonatal health in Africa: results using the Child Health and Nutrition Initiative method involving over 900 experts across the continent, https://doi.org/10.17605/OSF.IO/38GTU ^19^.

This project contains the following extended data files:

- MNCH Survey for AESA_AAS Scoring

Data are available under the terms of the Creative Commons Zero "No rights reserved" data waiver (CC0 1.0 Public domain dedication). 

## Notes

^1^ It is difficult to give the exact number because of snowballing and advertising beyond the notification of the African Academy of Sciences.
