# Supplementary material for: Research priorities in maternal and neonatal health in Africa: results using the Child Health and Nutrition Research Initiative method involving over 900 experts across the continent
Source: AAS Open Res. 2021 Feb 23;4:8. [Version 1] doi: 10.12688/aasopenres.13189.1 (PMC8204196; doi:10.12688/aasopenres.13189.1)
Supplement: Peer review history

# Peer review: Reviewer response for version 1

**DOI:** 10.21956/aasopenres.14299.r28587

**Published:** 2021-06-14

**Authors:** Ermel Johnson

**Affiliations:** 1 Department of Public Health and Research, West African Health Organisation, Bobo-Dioulasso, Burkina Faso

The article is interesting because it allows MNCH research actors to know the research priorities identified by the research actors themselves. The methodological approach used is participatory and replicable at all possible scales. The results allow to draw the attention of research community on the keys research priorities in MNCH.

 The authors lacked to involve policymakers in MNCH who have stake of the research and are the end users of research findings. This insufficiency should be stated in the study limits.

Is the work clearly and accurately presented and does it cite the current literature?

Yes

If applicable, is the statistical analysis and its interpretation appropriate?

Yes

Are all the source data underlying the results available to ensure full reproducibility?

Yes

Is the study design appropriate and is the work technically sound?

Yes

Are the conclusions drawn adequately supported by the results?

Yes

Are sufficient details of methods and analysis provided to allow replication by others?

Yes

Reviewer Expertise:

Implementation research; Knowledge Translation; Evidence Informed Decision Making; Health Policy and Systems; Maternal, Newborn and Child Health; Researh Governance

I confirm that I have read this submission and believe that I have an appropriate level of expertise to confirm that it is of an acceptable scientific standard.

# Peer review: Reviewer response for version 1

**DOI:** 10.21956/aasopenres.14299.r28588

**Published:** 2021-06-08

**Authors:** Narendra K. Arora

**Affiliations:** 1 The INCLEN Trust International, New Delhi, Delhi, India

The paper describes a research priority setting exercise for maternal and newborn health (MNH) undertaken in Africa in 2019 within the broader context of the SDGs. The authors have used the CHNRI method which is a widely accepted method for objectively and transparently identifying priorities using the ‘wisdom of the crowd’. The authors have followed the needful steps as have evolved for CHNRI over time and have come up with a promising list of priorities. The paper is also written in an easy to understand language with interesting tables. Nevertheless, there is a need for the authors to provide further information and to reconsider language syntax. Consequently, the current manuscript may be ' *approved with reservations*'.

 My specific comments and edit suggestions are as below:

**Pg 3**. “The Steering Committee constituted by the Grand Challenges Africa programme at the African Academy of Sciences oversaw the CHNRI exercise in 2019 for MNH in Africa."

Comment:

- **Please provide details of the composition of the Steering Committee. Were these exclusively from Africa?**

**Pg 3.** “ *i. The context of the exercise is defined”*

Comment:

- **Was a time frame also defined for the priority setting exercise context or was it set at 2030 (SDG)? **
- **There are likely to be between-and-within-country differences for the public health situation and program priorities in Africa. How were these taken into consideration while defining the context of this exercise?**

**Pg 3. “**Over 7001 experts, identified through a database held by the AAS, and through a literature review and snowballing, were invited to submit research ideas.”

Comment:

- **Please provide details of the database. Who were these experts and what was the criteria for inclusion in the database? Were of them MNH experts?**

**Pg 4.** “online survey from 251 experts using Survey Monkey (see *Extended data*19).”

Comment:

- **Also specify the response rate (%) within parentheses.**

**Pg 4.** “a cut-off score of > 4.25 was used that selected”.

Comment:

- **How was this cut-off identified?**

**Pg 4.** “selected 46 top research options to be presented for scoring by the larger group of experts”.

Comment:

- **Please provide break-ups according to the four grand challenge categories and also according to the 4D categories here (in-text) as well.**

**Pg 4.** “ *iv. External stakeholders weigh the criteria - *A workshop was held in June 2019”.

Comment:

- **What was the objective of this workshop? Was this an international workshop with participants from different countries in Africa or did it also have international participants? Were the participants from different organizations?**

**Pg 4.** “public and private sector, donors, civil society organizations, clinicians, and academics. A total of 42 stakeholders determined by availability but balanced by region, discipline, expertise, and gender participated in the weighting exercise. Stakeholders”.

Comment:

- **If the authors have the information, they are requested to provide the frequency for each stakeholder category mentioned.**
- **Does 'region' mean 'region within Africa' or is it ‘global regions’ (as used by WHO)?**

**Pg 4.** “ weight(criterion 1)= [(Σscores criterion 1ncriterion 1)10/]*4”.

Comment:

- **Please check if the formula is correctly mentioned or not**

**Pg 4.** “Survey Monkey was used to circulate the list of 46 research questions to the wider group of experts.”

Comment:

- **What was the number of experts approached?**

**Pg 4.** “ **Table 1.** **Description of CHNRI criteria and weights.”**

Comment:

- **The criteria 'Disease burden reduction' and 'Potential impact and equity' (especially sub-criteria (iii)) are likely to correlate in resource constrained African setting. Could this have affected the overall ranking of the priorities?**

**Pg 5.** “A total of 319 experts from across all regions (North, South, East, and West) in Africa scored the research questions in the 2-hour survey where questions were randomized and presented to scorers in a different order. Invited experts were top publishing authors from each country in MNCH from 2015 to 2019. Table 2 contains individual and demographic characteristics

 for each scorer, including country of residence, gender, age, and area of specialization. Duplicate responses were removed and scorers included in the analysis if they scored at least one full research question. A total of 195 scorers filled the survey completely (e.g. entered a score for every criterion for each research question).”

Comment:

- **Please specify participation rate.**
- **The table shows that 79 experts i.e., almost 25% were residing outside Africa. Were these experts belonging to Africa (diaspora) or non-African researchers?**
- **Do we have a break-up of what broad specialty these experts belonged to - basic science, clincial, socio-behavioral/ public health science?**
- **How were the 'top' publishing authors identified from each country? Was it through a structured search or nomination, and was there a percentile cut-off to identify the 'top' authors?**
- **Almost a third of the scorers were under 40 years age. So, possibly the criteria for 'top' publishing authors may have been beyond the number of publications. Please clarify.**
- **Were the 'top' publishing authors identified after stratifying for sectors? Africa has 54 countries - it is unlikely that equal number of experts were identified from 'each' country. Please elaborate on representation or rectify the sentence.**
- **Please clarify on how the duplicates were removed – if the scorers were allowed to take the survey more than once, was the response for the first attempt considered or the last one?**

**Pg 5.** “Three of the top ten research priorities were within the grand challenge ‘Better care at birth,’ two were in ‘Better care during pregnancy,’ four were in ‘Better hospital care of sick newborns,’ and one was in ‘better postnatal care for women and their newborns.’”

Comment:

- **Please arrange these in descending order of frequency for ease of understanding.**

**Pg 7.** “Develop and evaluate strategies for improved utilization¬†of Kangaroo Mother Care (KMC)¬†at the community level:”

Comment:

- **Typo-graphical error (‘**¬†’) **. Please rectify.**

**Pg 10.** “to changes at the meso-level of sub-district and district decision-making23,24 for sustainable change at the primary healthcare level. rom”

Comment:

- **Typographical error (‘. rom’). Please rectify.**

**Pg 10.** “should be adequately informed of key critical information as they nurse their babies.”

Comment:

- **What ‘key critical information’ should be informed?**

**Pg 10.**  “it is important to recognize their collective voice when putting together research priorities to support the drive to achieve the SDG’s. The MNH group at the African Academy of Sciences believe that this is the most rigorous process yet to highlight the MNH research and development priorities for Africa with the participation of over 900 MNH experts and practitioners.”

Comment:

- **Please remove the apostrophe for SDGs.**
- **900+ members were 'approached' but did the actual number of those who 'participated' could be less. Please verify.**

**Pg 10.** “R&D efforts should align with these needs to assist practitioners in achieving better maternal and neonatal outcomes. There is a need for better diagnostics and detection of high-risk newborn and maternal conditions to allow for these conditions to be managed within a slowly reacting healthcare system27–29. It is important to emphasize that maternal mortality should be considered as a human rights issue, to strengthen the African system’s jurisprudence and legal frameworks30,31 including multisectoral participation of interest groups like human rights non-governmental organizations31 for us to achieve the desired outputs.”

Comment:

- **Does 'practitioners' also include 'public health program managers and policy-makers'?**
- **These seem to be upstream issues for the priorities identified but could also be options for socio-behavioral and public health research. However, these do not show up on the top-most priority list of research options. Is this a limitation of the exercise or are the authors suggesting some possible ways forward beyond what the study findings report?**

**Pg 12.** “There is a need to align with groups within Africa when designing MNCH programmes on the continent.”

Comment:

- **This is logical but there is a need to explain (with reference) why this is necessary.**

**Pg 12.** “The AAS/AESA built a database of about 8,000 MNH commentators from peer-reviewed journals who were invited by email to participate in this survey. Only 10% of this number responded and interacted with the MNH working group for Africa. This provides for a selection bias with only those driven to respond able to complete the 2-hour survey.”

Comment:

- **Please expand abbreviations on first use.**
- **Was this for the scoring? 10% of 8000 i.e. 800 responded and of whom 319 participated in the survey, right? Please clarify.**
- **Could the authors please elaborate on how this self-selection bias could have impacted the study results.**

**Pg 12.** “only managed to do a sub-analysis for East Africa, West Africa and the diaspora commentators.”

Comment:

- **Please clarify if these were experts of international origin or African diaspora?**

**Pg 12.** “A repeat exercise would be required if implementation is delayed.”

Comment:

- **'Delayed' beyond what timelines? The sentence can be modified to *‘Despite the heightened focus on the COVID-19 pandemic and consequent need for careful allocation of depleted resources, the research priorities identified by this extensive exercise should help to bring focus on the most important undercurrent MNH issues in Africa, and serve as the background to future prioritization exercises.’***

**Pg 12.** “This work establishes a maternal and neonatal health research priority list for Africa, covering the four grand challenge areas, in which science and research are crucial in accelerating implementation and developing innovations.”

Comment:

- **Please simplify this phrase or delete it.**

**Pg 12.** “The African Academy of Sciences through the Grand Challenges Africa and working with partners has implemented a maternal and neonatal healthcare innovations support programme based on these priorities. In addition to this, there is ongoing work on MNH research mapping exercise for Africa which should form a suitable base for the development of an MNH research network.”

Comment:

- **Please provide a reference, if available.**

**Pg 12.** “greater gender representation in MNH science and a consolidated platform for bridging the science policy gap in MNH.”

Comment:

- **This introduces a new dimension to the manuscript. Please delete or elaborate in the results and discussion sections (with reference) why this is important to achieve in Africa.**

**Pg 12.** “but anecdotal information points to reductions in MNH indicators due to changes to health”.

Comment:

- **Please delete the word 'indicators'.**

**Pg 12.** “This is, therefore, a call to action for capable multisectoral organizations to focus on these priority areas a”.

Comment:

- **Please also add ‘researchers’ along with multisectoral organizations.**

Is the work clearly and accurately presented and does it cite the current literature?

Yes

If applicable, is the statistical analysis and its interpretation appropriate?

Yes

Are all the source data underlying the results available to ensure full reproducibility?

Yes

Is the study design appropriate and is the work technically sound?

Yes

Are the conclusions drawn adequately supported by the results?

Partly

Are sufficient details of methods and analysis provided to allow replication by others?

Partly

Reviewer Expertise:

Public Health, Immunization & Child Health

I confirm that I have read this submission and believe that I have an appropriate level of expertise to confirm that it is of an acceptable scientific standard, however I have significant reservations, as outlined above.

# Peer review: Reviewer response for version 1

**DOI:** 10.21956/aasopenres.14299.r28589

**Published:** 2021-06-07

**Authors:** Shiyam Sunder Tikmani

**Affiliations:** 1 Aga Khan University, Karachi, Pakistan

I must commend the African Academy of Sciences Maternal and Neonatal Health Working Group for Africa on their outstanding endeavor. Maternal and newborn mortality is high in South Asia and Africa, and the causes of death include infections, pregnancy complications, malnutrition, and other factors.

 Overall, the paper was very nicely written.

**Abstract:**

- Very well written and a good summary of the paper.

**Introduction:**

- Very well written.

**Methods:**

- Apart form snowball, how experts were selected is not clear.
- Why weights applied?
- Plan of analysis was not described.
- What is the purpose of sub-analysis? Are MNH priorities different according to geography (for example East vs. west Africa)?

**Results:**

- What is the age and gender distribution of the experts who participated in this exercise?

**Discussion:**

- What are the limitations of the study?

Is the work clearly and accurately presented and does it cite the current literature?

Yes

If applicable, is the statistical analysis and its interpretation appropriate?

Partly

Are all the source data underlying the results available to ensure full reproducibility?

Partly

Is the study design appropriate and is the work technically sound?

Yes

Are the conclusions drawn adequately supported by the results?

Yes

Are sufficient details of methods and analysis provided to allow replication by others?

Yes

Reviewer Expertise:

Maternal and Child Health

I confirm that I have read this submission and believe that I have an appropriate level of expertise to confirm that it is of an acceptable scientific standard.
